# Complications After Percutaneous Pedicle Screw Fixation for the Treatment of Unstable Spinal Metastases

**DOI:** 10.1245/s10434-016-5156-9

**Published:** 2016-03-10

**Authors:** Anne L. Versteeg, Jorrit-Jan Verlaan, Paul de Baat, Tim U. Jiya, Agnita Stadhouder, Carel H. Diekerhof, Guido B. van Solinge, F. Cumhur Oner

**Affiliations:** Department of Orthopedic Surgery, University Medical Center Utrecht, Utrecht, 3584CX The Netherlands; Department of Orthopedic Surgery, Catharina Hospital, Eindhoven, The Netherlands; Department of Orthopedic Surgery, VU University Medical Center, Amsterdam, The Netherlands; Department of Orthopedic Surgery, St. Elisabeth Hospital, Tilburg, The Netherlands; Department of Orthopedic Surgery, Isala, Zwolle, The Netherlands

## Abstract

**Background:**

Complications after surgical stabilization for the treatment of unstable spinal metastases are common. Less invasive surgical (LIS) procedures are potentially associated with a lower risk of complications; however, little is known regarding the complications after LIS procedures for the treatment of spinal metastases. Our primary objective was to determine the characteristics and rate of complications after percutaneous pedicle screw fixation (PPSF) for the treatment of mechanically unstable spinal metastases. The secondary objective was to identify factors associated with the occurrence of complications and survival.

**Methods:**

A retrospective multicenter cohort study of patients who underwent PPSF between 2009 and 2014 for the treatment of unstable spinal metastases was performed. Patient data pertaining to demographics, diagnosis, treatment, neurologic function, complications, and survival were collected.

**Results:**

A total of 101 patients were identified, 45 men (45 %) and 56 women (55 %) with a mean age of 60.3 ± 11.2 years. The median operating time was 122 (range 57–325) minutes with a median blood loss of 100 ml (based on 41 subjects). Eighty-eight patients (87 %) ambulated within the first 3 days after surgery. An overall median survival of 11.0 (range 0–70) months was observed, with 79 % of the patients alive at 3 months after treatment. Eighteen patients experienced a total of 30 complications; nonsurgical complications were the most commonly encountered. Prolonged operating time was independently associated with an increased risk of complications.

**Conclusions:**

A complication rate of 18 % was found after PPSF for unstable spinal metastases. Potential advantages of less invasive treatment are limited blood loss and high early ambulation rate.

The life expectancy of patients diagnosed with metastatic disease can vary from several weeks or months for patients with unfavorable aggressive primary tumors with bone and visceral involvement to several years for patients with exclusively bone metastases, with the spinal column being the most common location for bone metastases.^1^–^3^ Radiotherapy has been the standard of care for the treatment of symptomatic spinal metastases.^4^,^5^ However, if spinal metastases compromise the mechanical integrity of the spine, surgical stabilization is the preferred treatment option, followed by radiotherapy for local tumor control.^6^

Surgical stabilization has traditionally been performed through open procedures necessitating extensive soft tissue dissection associated with significant blood loss, lengthy hospital stays, and a substantial risk of complications.^7^ Invasive procedures are—considering the limited life expectancy and comorbidities of most patients with spinal metastases—often undesirable and unfeasible.^8^

Advancements in surgical techniques have led to the development of the concept of less invasive surgical (LIS) procedures with the aim of achieving the same clinical results with less morbidity related to surgical approach.^8^ Benefits of less invasive techniques include decreased blood loss, less postoperative pain, and shortened recovery time.^8^ Moreover, LIS procedures allow earlier initiation of postoperative adjuvant treatments due to faster wound healing.^9^ The ability to perform LIS procedures for the treatment of unstable spinal metastases may enable surgeons to offer surgical intervention to patients who were not deemed to be candidates for conventional open surgery.^8^

LIS procedures have been shown to be safe and have resulted in good clinical outcomes in patients with traumatic and degenerative spinal disorders.^10^–^12^ Only a few studies have investigated the clinical outcome of less invasive techniques for the treatment of spinal metastases, with improved pain scores and functional status being reported.^13^–^16^ However, a paucity of literature exists regarding the safety of less invasive surgery for the treatment of symptomatic spinal metastases. Therefore, the primary objective of this study was to determine the characteristics and rate of complications after percutaneous pedicle screw fixation (PPSF) for the treatment of mechanically unstable spinal metastases. The secondary objective was to identify factors associated with the occurrence of complications and survival.

## Methods

A multicenter retrospective observational cohort study of patients who underwent PPSF for the treatment of unstable spinal metastases was performed. The local institutional review board approved the research protocol. Patients were eligible for inclusion if they had histologic proof of malignancy (including multiple myeloma) and were treated with PPSF, with or without cement augmentation, between January 2009 and December 2014. An (impending) unstable pathologic fracture and/or intractable mechanical pain due to an impending pathologic fracture were indications for surgical intervention. In addition, a life expectancy of at least 3 months, as assessed by the referring oncologist, was required. Assessment of the degree of spinal instability was based on the clinical experience of the surgeon and/or the recent spinal instability neoplastic score.^17^ To enhance the homogeneity of the procedure and study population, patients were excluded if they were diagnosed with a primary spinal tumor or if additional minimal-access spine surgery (e.g., mini open decompression, laminectomy) was performed.

Data pertaining to demographics, primary tumor diagnosis, surgical treatment, neurologic status, performance status, complications, and survival were collected from medical charts and institutional databases. Government databases were accessed to retrieve information about vital statistics. The definitions and scales of the outcome parameters are listed in Table 1.

**Table 1:** Definitions of outcome parameters

Parameter	Definition	Scale/unit	Time points
Neurologic status	Degree of neurologic deficit	ASIA scale^24^	Pre- and postoperative, first follow-up visit
Performance status	Level of daily functioning	Karnofsky^25^	Preoperative
Consultation time	Time from first surgical consultation until date of surgery	Days	NA
Ambulatory function	Able to walk at least 4 steps^20^	Days	NA
Blood loss	Estimated blood loss	Milliliters	NA
Complication	Any unexpected and undesirable medical event that required additional intervention or monitoring	NA	Peri- and postoperative
Operating time	Time from first incision until wound closure (“skin to skin”)	Minutes	NA
Hospital stay	Date of surgery until date of discharge	Days	NA
Follow-up time	Date of surgery until date of death	Months	NA

*ASIA* American Spinal Injury Association

Continuous data were described using mean, median, standard deviation, and range. Frequencies were used to describe categorical data. Univariate logistic regression was performed to identify predictive factors for the occurrence of complications. Linear regression analysis was conducted to determine variables related to length of stay. The Kaplan–Meier analysis and log-rank tests were used to investigate factors that influenced survival, followed by multivariate Cox regression analysis to determine the impact of the variables. *P* < 0.05 defined significance. IBM SPSS Statistics 21.0 for Windows was used for the analysis (IBM, Armonk, NY, USA).

## Results

### Demographics

A total of 101 patients were identified in the five participating centers; 45 patients were male (45 %) and 56 were female (55 %), and mean age was 60.3 ± 11.2 years. Patient characteristics are listed in Table 2. Breast cancer (25 %) and multiple myeloma (25 %) were the most frequent primary tumors. Fifty percent of the patients had exclusively metastatic bone disease, 42 % had bone and visceral metastases, and the remaining 8 % had bone and lymph node metastases. Ninety-four of the patients (93 %) were neurologically intact before surgery [American Spinal Injury Association (ASIA) E], six patients (6 %) had minimal motor impairment (ASIA D) without progression, and one patient (1 %) had severe motor impairment (ASIA C).

**Table 2:** Baseline characteristics

Characteristic	Value
Gender (*n* = 101)	
Female	56 (55 %)
Male	45 (45 %)
Age at surgery, years (*n* = 101)	60.3 (SD 11.2)
Primary tumor type (*n* = 101)	
Breast	25 (25 %)
Multiple myeloma	25 (25 %)
Lung	13 (13 %)
Kidney	10 (10 %)
Prostate	5 (5 %)
Other	23 (22 %)
Clinical presentation (*n* = 101)	
Back pain	53 (52 %)
Radicular pain	7 (7 %)
Combined radicular and back pain	20 (20 %)
Impending fracture without significant pain	21 (21 %)
Karnofsky performance status (*n* = 98)	
100 %	4 (4 %)
80–90 %	46 (47 %)
60–70 %	28 (28 %)
40–50 %	20 (21 %)
<30 %	0 (0 %)
Preoperative ASIA scale (*n* = 101)	
E	94 (93 %)
D	6 (6 %)
A/B/C	1 (1 %)

*ASIA* American Spinal Injury Association

### Operative Characteristics

Median time from first surgical consultation to surgical intervention was 9 (range 0–377) days. The most commonly treated areas were the thoracolumbar (T10–L2, *n* = 39) and thoracic (*n* = 38) regions, followed by the lumbar (*n* = 22) and lumbosacral (L4–S2, *n* = 2) regions. The median operating time was 122 (range 57–325) minutes; median blood loss was 100 (range 50–500) ml based on data available for 41 patients. Five or more vertebral bodies were bridged in 65 patients (64 %), four in nine patients (9 %), and three in 27 patients (27 %). Vertebroplasty was performed in six patients (6 %), kyphoplasty in 10 patients (10 %), and vertebral body stenting in 19 patients (19 %). Cement augmentation of pedicle screws was performed in three patients (3 %).

Eighty-seven percent of the patients (*n* = 88) were ambulatory within the first 3 days after surgery (median 1 day), and overall median length of hospital stay was 7 (range 1–43) days. Patients who experienced a complication had a median length of hospital stay of 11.5 days, which was significantly longer compared to a median length of hospital stay of 7 days for patients without complications (*P* = 0.002). The overall presence of complications (*P* = 0.003), the need for reoperation (*P* < 0.001), the presence of neurologic deterioration (*P* = 0.015), the presence of construct failure (*P* < 0.001), and the presence of nonsurgical complications (*P* = 0.026) were associated with increased length of hospital stay. The most common form of adjuvant treatment was postoperative radiotherapy in 56 patients (55 %). Fifteen patients (15 %) had received radiotherapy before surgery, 11 patients (11 %) received both pre- and postoperative radiotherapy, and 17 patients (16 %) did not receive additional radiotherapy.

### Complications

A total of 30 complications occurred (Table 3), with 18 patients experiencing at least one complication. Prolonged operating time (*P* = 0.041) was the only factor significantly associated with the occurrence of complications. Nonsurgical adverse events were common, including delirium (*n* = 3), pneumonia (*n* = 2), ileus (*n* = 1), urinary tract infection (*n* = 1), and bladder retention (*n* = 1). Furthermore, one patient developed a perioperative acute coronary syndrome. This patient was transported to the intensive care unit after surgery and died 3 days later of cardiac failure.

**Table 3:** Complications in 101 patients

Complication	*n* (%)
Superficial wound infection	2 (2)
Deep wound infection	2 (2)
Neurologic deterioration	
Transient deterioration	1 (1)
Surgical permanent deterioration	2 (2)
Secondary permanent deterioration	3 (3)
Construct failure	
Within 3 months	1 (1)
After 3 months	3 (3)
Malposition of screw	1 (1)
Reoperation	6 (6)
Other complications	9 (9)

A wound-healing disturbance occurred in four patients; two of these were deep wound infections. Four patients experienced construct failure; two patients had a pedicle screw pullout, with one of the two patients requiring a reoperation; and one patient had a broken pedicle screw (S1), causing pain and requiring reoperation 1.5 years after the index surgery. In one patient, secondary screw pullout occurred as a result of tumor progression, which required revision surgery within 1 month of the index surgery. After the second surgery, the patient developed a superficial wound infection and experienced neurologic deterioration due to tumor growth into the spinal canal; neither preoperative nor postoperative radiotherapy was administered. Local tumor progression was also the cause for neurologic deterioration in two other patients, both within 4 months after index surgery. Neurologic deterioration also occurred in two other patients; one experienced permanent complete paraplegia resulting from medial placement of a pedicle screw, and revision surgery was performed without postoperative neurologic improvement. Cement extravasation resulting in an incomplete spinal cord lesion (ASIA C), from which the patient recovered fully (ASIA E) after reoperation, was the cause in the other patient. A total of six patients (7 %) required revision surgery. One patient experienced transient neurologic deterioration immediately after surgery at the recovery unit but recovered spontaneously within 6 h. Neurologic status over time is displayed in Table 4.

**Table 4:** Neurologic status over time

ASIA score	Preoperative	Postoperative	Follow-up^a^
E	94	94	93
D	6	3	3
C	1	1	1
B	0	1	1
A	0	1	1

*ASIA* American Spinal Injury Association

^a^One patient died in hospital; last neurologic function was postoperative

### Survival

**Fig. 1:**
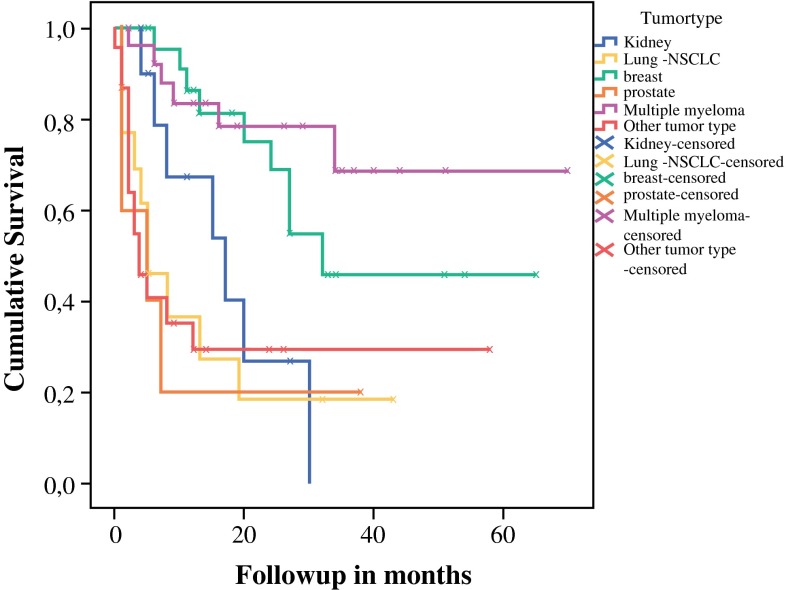
Kaplan–Meier survival curve. Differences in survival between different tumor types were tested by log-rank test, *P* < 0.001. Excluding multiple myeloma, *P* < 0.001

The overall median survival was 11.0 (range 0–70) months, with 39 patients (39 %) still alive in March 2015. Seventy-nine patients (78 %) were alive 3 months after surgery. Kaplan–Meier analysis revealed that patients with breast carcinoma and multiple myeloma had significantly better survival compared to other primary tumor types (Fig. 1). Univariate analysis demonstrated that lower performance status (*P* = 0.043), primary tumor type (*P* < 0.001), presence of node and/or organ metastases (*P* = 0.019), and no administration of postoperative chemotherapy (*P* = 0.007) negatively influenced 3-month survival. Using multivariate analysis only, the lack of administration of postoperative chemotherapy [*P* = 0.017, hazard ratio (HR) 5.8, 95 % confidence interval (CI) 1.79–18.77] was demonstrated to be independently associated with mortality within 3 months after surgery.

Overall survival was negatively influenced by older age (*P* < 0.001), primary tumor type (*P* < 0.001), and the presence of node and/or or visceral metastases (*P* < 0.001) in the univariate analysis. Multivariate analyses demonstrated that two factors were independently associated with impaired overall survival: a diagnosis of primary tumor type other than breast, prostate, lung, or renal carcinoma (*P* = 0.006, HR 3.94, 95 % CI 1.4–8.1), and the presence of lymph node and/or visceral metastases (*P* < 0.005, HR 2.9, 95 % CI 1.37–6.1).

## Discussion

Advancements in surgical techniques and implants have led to the development of LIS procedures. Thus far, studies reporting the clinical outcomes after minimally invasive procedures for the treatment of spinal metastases have been few.^13^–^16^ These studies reported promising clinical results in terms of decreased postoperative pain levels and early recovery of ambulatory function.^13^–^16^ To our knowledge, we present the largest cohort of patients who underwent PPSF, with or without cement augmentation, for the treatment of spinal metastases, and the first study with a specific focus on the characteristics and rate of complications. In addition, factors that could predict the occurrence of complications or influenced survival were analyzed.

This study demonstrated that 18 % of the patients experienced at least one complication, with an increased risk for complications with longer operation duration. Seven patients required revision surgery for construct failure, neurologic deterioration, or surgical debridement of a deep wound infection. Two different studies reporting on outcomes after PPSF, but without specific focus on complications, reported complication rates of 9 % (4 of 46) and 17 % (2 of 12).^13^,^14^ In comparison, several retrospective studies have investigated complications rates after open surgical procedures for the treatment of spinal metastases, with complication rates reported between 15 and 47 %.^7^ Furthermore, Dea et al. conducted a prospective study on adverse events after emergency spine surgery for spinal metastases.^7^ A complication rate of 76 % was found, with a mean of 1.8 adverse events per patient.^7^ Both prospective and retrospective studies report high complication rates after surgical intervention for spinal metastases.^7^ The high risk for complications does not only reflect the surgical demand of these procedures but also reflects the fragility of this patient category.^18^ Our reported complication rate of 18 % falls in the lower range of previously published complication rates, suggesting that PPSF for the treatment of spinal metastases may result in fewer complications compared to conventional open procedures. However, the retrospective design of this study may also account for this lower complication rate. Minor complications may not have been registered in the patient’s medical chart. In addition, only patients who underwent PPSF were included for analysis. This limits the direct comparison of complication rates between different surgical approaches. However, by including only patients who underwent isolated PPSF, we aimed to create homogeneity regarding the surgical procedure, thereby facilitating more accurate interpretation of the complications associated with PPSF. The multicenter research approach has resulted in a relatively large number of patients, thereby increasing the generalizability of the results. It should, however, be noted that PPSF, in regular practice, is also frequently combined with decompressive techniques as a LIS procedure.

Three (3 %) of our patients experienced neurologic deterioration caused by local tumor progression. This rate is similar to other studies reporting decreased ambulatory and/or neurologic function due to local disease progression resulting in spinal cord compression.^13^,^14^ Although this rate is relatively low, the impact of this complication on quality of life is substantial and is also associated with decreased survival rates.^19^,^20^ Symptomatic spinal cord compression is best treated with the combination of surgical decompression, stabilization, and radiotherapy.^21^ PPSF techniques can successfully be combined with mini open decompressive techniques in patients with symptomatic spinal cord compression. However, because the benefits of percutaneous surgical procedures, compared to conventional open techniques, quickly diminish when decompressive techniques are required as a result of the increased risk of complications, the presence of symptomatic spinal cord compression may be regarded as a relative contraindication for PPSF techniques.

Although LIS procedures have potential benefits, there are also some limitations. First, LIS procedures depend on accurate intraoperative visualization of the bony anatomy to minimize the risk of screw malposition and to prevent cement leakage. Second, the implants used in LIS procedures serve as an internal brace because bony fusion is not achievable with most LIS procedures.^11^,^16^ However, considering the limited life expectancy of most patients with spinal metastases, the main goal is to improve quality of life by stabilizing the spine rather than achieving fusion, as is the goal with traumatic fractures.^16^ Third, only limited sagittal correction can be achieved using current LIS procedures compared to an open procedure.^11^ It should be noted that the term “LIS procedure” does not encompass one surgical technique but rather is a surgical concept including a wide variety of surgical procedures, including minimal-access decompression of the spinal cord.

The most frequent complications in our study were neurologic deterioration (6 %) and revision surgery (6 %), with three patients requiring revision surgery as a result of neurologic deterioration. In contrast, studies reporting on adverse events after open surgical procedures report infection (including wound infections), pneumonia, and hematoma as frequent complications.^7^,^18^ Four of our patients experienced a wound complication consisting of two deep and two superficial wound infections. No excessive blood loss or hematomas were reported. The differences in complication types between open surgical procedures and percutaneous procedures can be explained by the difference in surgical approach, with PPSF having several potential advantages over the open approach. First, PPSF is performed through small stab incisions. The combined total length of the incisions may be the same or longer compared to open surgery, but the smaller incisions limit soft tissue dissection, minimize blood loss, decrease postoperative pain, and decrease the risk of wound-healing disturbances. Less postoperative pain also results in less analgesics use, earlier ambulation, and shorter hospital stay. This study reported a median length of hospital stay of 7 days, and 78 % of the patients were ambulatory within the first 3 days after surgery. A significant difference was found between the length of stay of patients with and without complications. Finally, PPSF is associated with less blood loss, with a median blood loss of 100 ml in the present study. Significant blood loss has been associated with lengthy hospital stays as well as increased morbidity and mortality rates.^8^ Furthermore, significant blood loss often requires blood transfusions, which are associated with immunosuppression and a subsequent increased risk of infection and disease progression.^22^

Most of the patients who require surgical intervention for the treatment of spinal metastases are subsequently treated with radiotherapy for local control. In addition, chemotherapy and/or immunotherapy are often initiated as systemic treatment. Improved wound healing caused by the smaller incisions allows for earlier initiation of postoperative adjuvant therapies.^9^ Earlier initiation or continuation of adjuvant therapies is important in the palliative phase to maximize tumor control.^23^ Fewer complications and shortened rehabilitation time with LIS procedures may also enable surgical intervention for patients who were not considered to be good surgical candidates for extensive open surgery on the bases of their life expectancy and physical status.^8^

In conclusion, to our knowledge, this is the first study to specifically focus on complications after PPSF for the treatment of spinal metastases. A complication rate of 18 % was found, suggesting that PPSF may lead to fewer complications compared to complication rates of open surgical procedures that have been reported in the literature.^7^ Prolonged operating time was demonstrated to be associated with an increased risk of complications. In addition, the absence of postoperative administration of chemotherapy was associated with mortality at 3 months after surgery. Potential advantages of LIS procedures consist of decreased need for blood transfusions, decreased need for analgesics, early ambulatory function, shorter hospital stay, and earlier initiation of postoperative adjuvant therapies. Future prospective studies are needed to improve our insight in the frequency and types of complications after different types of LIS procedures.
